# Degeneration Directory: a multi-omics web resource for degenerative diseases

**DOI:** 10.1093/procel/pwad066

**Published:** 2023-12-28

**Authors:** Haoteng Yan, Changfa Lu, Chenyang Lan, Si Wang, Weiqi Zhang, Zan He, Jinghao Hu, Jiaqi Ai, Guang-Hui Liu, Shuai Ma, Yuanchun Zhou, Jing Qu

**Affiliations:** State Key Laboratory of Membrane Biology, Institute of Zoology, Chinese Academy of Sciences, Beijing 100101, China; State Key Laboratory of Stem Cell and Reproductive Biology, Institute of Zoology, Chinese Academy of Sciences, Beijing 100101, China; Key Laboratory of Organ Regeneration and Reconstruction, Institute of Zoology, Chinese Academy of Sciences, Beijing 100101, China; Advanced Innovation Center for Human Brain Protection, and National Clinical Research Center for Geriatric Disorders, Xuanwu HospitalCapital Medical University, Beijing 100053, China; Aging Translational Medicine Center, International Center for Aging and Cancer, Beijing Municipal Geriatric Medical Research Center, XuanwuHospital, Capital Medical University, Beijing 100053, China; Computer Network Information Center, Chinese Academy of Sciences, Beijing 100190, China; Computer Network Information Center, Chinese Academy of Sciences, Beijing 100190, China; Advanced Innovation Center for Human Brain Protection, and National Clinical Research Center for Geriatric Disorders, Xuanwu HospitalCapital Medical University, Beijing 100053, China; Aging Translational Medicine Center, International Center for Aging and Cancer, Beijing Municipal Geriatric Medical Research Center, XuanwuHospital, Capital Medical University, Beijing 100053, China; The Fifth People’s Hospital of Chongqing, Chongqing 400062, China; Aging Biomarker Consortium, Beijing 100101, China; Aging Biomarker Consortium, Beijing 100101, China; CAS Key Laboratory of Genomic and Precision Medicine, Beijing Institute of Genomics, Chinese Academy of Sciences and China NationalCenter for Bioinformation, Beijing 100101, China; University of Chinese Academy of Sciences, Beijing 100049, China; Sino-Danish College, University of Chinese Academy of Sciences, Beijing 101408, China; Advanced Innovation Center for Human Brain Protection, and National Clinical Research Center for Geriatric Disorders, Xuanwu HospitalCapital Medical University, Beijing 100053, China; Aging Translational Medicine Center, International Center for Aging and Cancer, Beijing Municipal Geriatric Medical Research Center, XuanwuHospital, Capital Medical University, Beijing 100053, China; Advanced Innovation Center for Human Brain Protection, and National Clinical Research Center for Geriatric Disorders, Xuanwu HospitalCapital Medical University, Beijing 100053, China; Aging Translational Medicine Center, International Center for Aging and Cancer, Beijing Municipal Geriatric Medical Research Center, XuanwuHospital, Capital Medical University, Beijing 100053, China; Advanced Innovation Center for Human Brain Protection, and National Clinical Research Center for Geriatric Disorders, Xuanwu HospitalCapital Medical University, Beijing 100053, China; Aging Translational Medicine Center, International Center for Aging and Cancer, Beijing Municipal Geriatric Medical Research Center, XuanwuHospital, Capital Medical University, Beijing 100053, China; State Key Laboratory of Membrane Biology, Institute of Zoology, Chinese Academy of Sciences, Beijing 100101, China; Key Laboratory of Organ Regeneration and Reconstruction, Institute of Zoology, Chinese Academy of Sciences, Beijing 100101, China; Advanced Innovation Center for Human Brain Protection, and National Clinical Research Center for Geriatric Disorders, Xuanwu HospitalCapital Medical University, Beijing 100053, China; Aging Translational Medicine Center, International Center for Aging and Cancer, Beijing Municipal Geriatric Medical Research Center, XuanwuHospital, Capital Medical University, Beijing 100053, China; Aging Biomarker Consortium, Beijing 100101, China; University of Chinese Academy of Sciences, Beijing 100049, China; Beijing Institute for Stem Cell and Regenerative Medicine, Beijing 100101, China; Institute for Stem Cell and Regeneration, Chinese Academy of Sciences, Beijing 100101, China; State Key Laboratory of Membrane Biology, Institute of Zoology, Chinese Academy of Sciences, Beijing 100101, China; Key Laboratory of Organ Regeneration and Reconstruction, Institute of Zoology, Chinese Academy of Sciences, Beijing 100101, China; Aging Biomarker Consortium, Beijing 100101, China; University of Chinese Academy of Sciences, Beijing 100049, China; Beijing Institute for Stem Cell and Regenerative Medicine, Beijing 100101, China; Institute for Stem Cell and Regeneration, Chinese Academy of Sciences, Beijing 100101, China; Computer Network Information Center, Chinese Academy of Sciences, Beijing 100190, China; State Key Laboratory of Stem Cell and Reproductive Biology, Institute of Zoology, Chinese Academy of Sciences, Beijing 100101, China; Key Laboratory of Organ Regeneration and Reconstruction, Institute of Zoology, Chinese Academy of Sciences, Beijing 100101, China; Aging Biomarker Consortium, Beijing 100101, China; University of Chinese Academy of Sciences, Beijing 100049, China; Beijing Institute for Stem Cell and Regenerative Medicine, Beijing 100101, China; Institute for Stem Cell and Regeneration, Chinese Academy of Sciences, Beijing 100101, China

## Background of database

Organ degeneration refers to the gradual decline in organ function and structure deterioration that occurs during aging, which represents the greatest risk factor for various degenerative diseases, including cardiovascular diseases, neurodegenerative diseases, and osteoarthritis, etc. (Aging Biomarker et al., 2023; Becker et al., 2018; Cai et al., 2022). Billions of people around the world suffer from degenerative diseases, and these impose an outsized burden on the global healthcare system (Collaborators, 2019; Wolters et al., 2020). Yet, the majority of research efforts directed toward degenerative diseases is focused on a single disease, such as Parkinson’s disease, arthritis, or diabetes, which prevents a deeper understanding of the nature of organ degeneration and their link to the degenerative diseases (Guo et al., 2022). Thus, the establishment of a comprehensive database focusing on degenerative diseases and organ degeneration is therefore of the utmost urgency. A comprehensive collection of multi-level omics data encompassing multiple degenerative diseases would facilitate comparisons between aging and degenerative diseases, but more importantly, also support collaborations between research scientists and clinicians to accelerate the development of therapeutic strategies and aging interventions preventing or delaying disease onset (Liu et al., 2022; Jing et al., 2023).

High-throughput omics technologies, such as spatial transcriptomics, single-cell transcriptomics, and epigenomics, have played an important role in enabling scientists to analyze essential physiopathological processes associated with aging and degenerative diseases (Risbud and Shapiro, 2014; Sun et al., 2020; Tiklova et al., 2020; Yan et al., 2023; Zhou et al., 2022;Yan et al., 2021). To delineate degenerative diseases at the molecular level, it is imperative to establish a comprehensive degenerative disease database equipped to store, normalize, integrate, and analyze multi-omics sequencing data. By comparing cellular and molecular alterations associated with organ degeneration in different diseases, we stand to gain a deeper understanding of shared mechanisms underlying aging and degenerative diseases. Moreover, such an approach has the potential to facilitate the identification of key events that promote organ degeneration prior to disease onset and to catalyze the development of relevant interventions or therapeutic approaches that advance human health (Carelock et al., 2023). Consequently, the systematic integration of multi-omics sequencing data represents an essential step to advance the development of treatment for degenerative diseases.

Presently, existing databases that contain data related to degenerative diseases include ADNI (Petersen et al., 2010), scREAD (Jiang et al., 2020), AD Knowledge Portal (Greenwood et al., 2020), Gene4PD (Li et al., 2021), and ALSoD (Abel et al., 2013). These databases provide data resources and platforms for scientists to study disease progression, but substantial gaps that require attention and resolution remain. First, most of these databases primarily focus on a single disease, such as Alzheimer’s disease, precluding a comprehensive understanding of the molecular relationships and common/specific features across a broader spectrum of degenerative diseases. Second, these databases are often restricted to a single tissue and omics data, which hinders the broader discovery of previously overlooked but potentially essential molecular changes associated with organ degeneration. Consequently, exploring genetics and gene expression profiling across different diseases and omics in multiple platforms becomes very challenging. Overcoming these limitations is crucial to gain insights into the key molecular events underlying organ degeneration and degenerative diseases, explore their clinical and interventional potential and ultimately advance the fields of aging biology and life medicine.

Given the increasing accumulation of degenerative disease-related studies containing a broad spectrum of omics, it is imperative for the broad field to aggregate, normalize, and analyze such data into a database (Kang et al., 2022). Such a resource should enable comparative analysis of degeneration and diseases temporally and spatially at the molecular level across multiple omics dimensions and multiple organ degenerative processes. To this end, we present the degeneration directory (DD), a multi-omics web resource for the exploration of degenerative diseases. The DD is composed of six omics modules including transcriptome, single-cell transcriptome, spatial transcriptome, epigenome, proteome, and microbiome. The utilization of DD holds great significance for public health, which provides extensive multi-omics data and information on various degenerative diseases, contributing to the enhancement of disease comprehension, early diagnosis, and intervention. Our database will thus provide a valuable resource for the broader life scientist and clinical community.

### Database content

**Figure 1.**
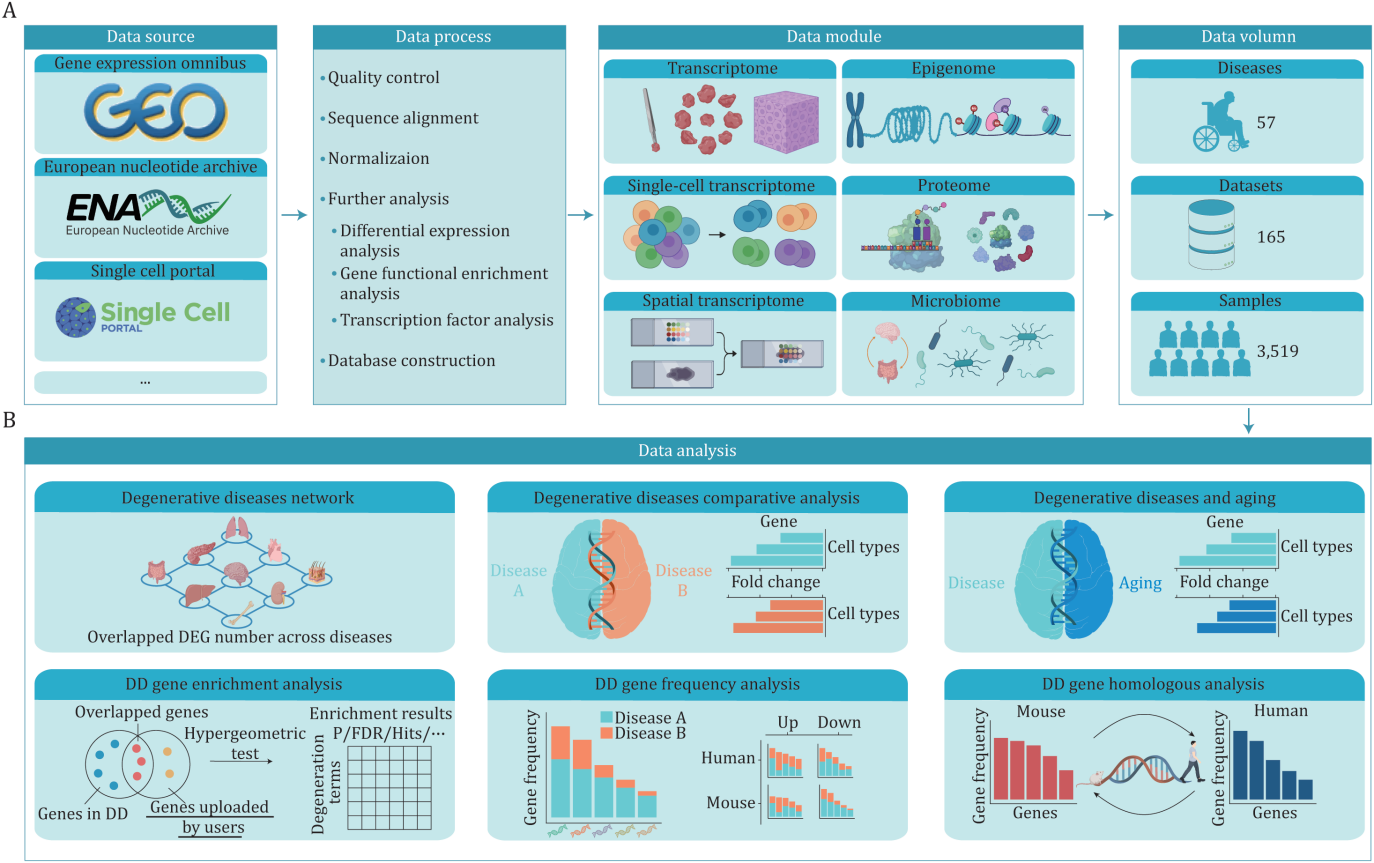
Overview of the DD database. (A) The flow chart of DD construction. (B) Based on omics data, six data analysis tools are provided to facilitate user explorations of molecular changes associated with degeneration.

The DD aims to collect omics sequencing data related to multiple organ degenerative diseases in humans and mice, including neurodegenerative diseases, osteoarthritis, and other diseases related to degeneration. The DD now comprises six types of omics data, including transcriptome, single-cell transcriptome, spatial transcriptome, epigenome, proteome, and microbiome. Multiple analysis and visualization tools are accessible to help users explore molecular changes underlying degeneration (Fig. 1). In summary, our database currently includes over 57 diseases, 3,519 samples, and 165 datasets. Thus, our database provides a convenient data resource for scientists to explore novel therapeutic targets, with the potential to make valuable contributions to public health.

### Transcriptome

Bulk-level RNA-seq captures global changes in gene expression levels associated with organ degeneration and disease progression (Pandey et al., 2022). In the DD transcriptome module, two main types of data are accessible, including lists of differentially expressed genes (DEGs) and functional annotated biological pathways during disease progression. For example, the expression of *CD3D* and *CD3E* is increased in various diseases subtypes of glomerulonephritis and diabetes, which suggests that T-cell-mediated cellular immunity may play an important role in disease progression, offering a new perspective for potential treatment in the context of glomerulonephritis and diabetes. On the far right of each differential gene data entry, a boxplot can visually present changes in gene expression upon hovering the mouse over it. The transcriptome module contains public transcriptome data of more than 32 organ degeneration-related diseases from 895 samples and 84,558 DEGs. This module is continuously updated to include more high-quality datasets.

### Single-cell transcriptome

Single-cell sequencing, an emerging transcriptome technology, has contributed toward our understanding of a variety of life processes, including organ degeneration, at unprecedented resolution (Kuchroo et al., 2023; Leng and Pawelec, 2022; Rickner et al., 2022). The DD single-cell transcriptomics module collects transcriptomics data associated with organ degeneration or degenerative diseases and documents cell type-specific changes systematically. Users can click on the dataset number to further browse the details and data of each dataset. This module covers 25 diseases, 2,730,240 cells, and 409,155 disease-related DEGs. The main data types include dataset metadata, cell type-specific markers, disease-associated DEGs, and key transcription factors. For example, the gene expression of *MAPT*, a gene encoding Tau protein, is commonly increased in oligodendrocytes among patients with Alzheimer’s disease. The tangles of this protein have been recognized as one of the important pathological characteristics of neurological diseases. Molecular changes at the cell-type level will inform the design of screens and suitable cell models for drug development. In addition, users can explore changes in the number of cells and DEGs within the visualization module. All data presented on the page can be downloaded without any restriction in the download module.

### Spatial transcriptome

Sequencing and image-based spatial transcriptomics techniques have been applied to a variety of degenerative diseases (Chen et al., 2020; Navarro et al., 2020). Spatially resolved transcriptomics providing gene expression profiles with positional information is helpful in deciphering the spatial molecular distribution in tissues during disease progression. This module focuses on the spatial expression of RNA and contains more than 185 slices and 341,467 spots data in Alzheimer’s disease, osteoarthritis, and periodontitis. We developed an online visualization tool that allows users to freely explore changes in gene expression levels across slices. In the future, the optimized visualization will be updated to improve the contextual understanding.

### Epigenome

Accumulating evidence indicates that epigenetic mechanisms, including DNA methylation, histone modification, and others, contribute to the pathogenesis of multiple degenerative diseases. (Hampel et al., 2021; Nativio et al., 2020). In the epigenome module, we included epigenetic data from more than 330 samples of 10 diseases including Alzheimer’s disease and Parkinson’s disease, with data types including ChIP-seq, ATAC-seq, etc. For example, the gain of H3K27ac and H3K9ac marks has been reported in Alzheimer’s disease and after selecting samples and genes of interest, users can browse the signal values of corresponding genomic regions in this module.

### Proteome

Changes in protein abundance are an important basis for the occurrence and progression of degenerative diseases. For example, proteomic studies of brain tissue from patients with neurodegenerative diseases have shown that phosphorylation of the tau protein is the hallmark of neurodegenerative disease (Bai et al., 2020). The proteome module focuses on protein abundance changes during multiple diseases and includes more than 567 samples with 27,544 differentially abundant proteins from brain tissues, cerebro-spinal fluid, and plasma. Users can query changes in any protein of interest by inputting the gene name or the primary accession number in the protein UniProt database. For example, the abundance of the Caspase 14 protein, encoded by gene *CASP14*, is increased in the plasma of patients with Parkinson’s disease and could serve as a potential disease biomarker. The proteome information will promote the development of biomarkers and therapeutic interventions.

### Microbiome

Gut microbes affect other organs including the central nervous system through mechanisms such as metabolites and the gut-brain axis (Aho et al., 2019; Cryan et al., 2019). The relationship between multiple diseases and the gut microbiota has been widely reported (Liu et al., 2019; Zhang et al., 2020). In this module, we analyze changes in the abundance of gut microbes associated with degenerative diseases. Currently, 16S rDNA and metagenomic data from more than 1,200 patients have been included. For example, the abundance of the anaerobic bacteria *Blautia* is increased in the gut microbiota of patients with Parkinson’s disease, which provides us with the opportunity to further investigate the role of gut microbes in neurodegenerative diseases. Users can quickly browse and query the abundance changes of related microorganisms on the database page, and search hierarchically according to different levels such as phylum, order, family, genus, and species.

## Database usage

### Degenerative diseases network

More and more evidence shows that there is a common molecular basis in the process of organ degeneration (Dopazo et al., 2016; Liu et al., 2020; Stockwell et al., 2020). Exploring the degree of omics similarity between degenerative diseases is therefore highly significant for furthering our understanding of disease mechanisms, as such efforts will help identify essential changes associated with organ degeneration and inform the development of drugs and treatments for multiple disease targets. By comparing the similarity of differentially expressed genes (DEGs) in different disease processes, we developed a network landscape in which users can explore which diseases have more similar transcriptome changes, degree of omics similarity, and potential molecular connections among diseases. Users can select their disease of interest and then highlight the corresponding data. In addition, the list of DEGs used to build the disease network is also displayed below the network diagram. This module compares and analyzes the changes among different organ degenerative lesions and degenerative diseases, aiming to reveal the potential connections and differences between disease-associated lesions in various organs.

### Degenerative diseases comparative analysis

To identify molecular changes at the cell type level between organ degenerative diseases, a separate module was developed. Users can compare any two degenerative diseases by simply entering a gene of interest, and the DEGs of all cell types in the two selected diseases will be displayed. A log-normalized fold-change value greater than 0 indicates an increase in expression during disease progression, whereas a value less than 0 indicates a decrease. For example, the *APOE* gene showed a more consistent pattern in Parkinson’s disease and Huntington’s disease with increased expression levels in microglia and decreased expression levels in astrocytes. These findings offer potentially valuable insights for the exploration of targets for disease intervention. This module comparatively analyzes molecular changes in cell types between degenerative diseases, aiming to reveal potential links and differences across different degenerative diseases.

### Degenerative diseases and aging

A cumulative body of work supports that risk factors and molecular mechanisms overlap in organ aging and tissue degenerative processes, and analyzing the potential molecular correlation between organ aging and degenerative diseases is therefore of high importance (Rubinsztein et al., 2011; Stevnsner et al., 2002). In this module, we include the DEGs associated with organ aging from the Aging Atlas database (Aging Atlas, 2021) to compare organ aging with human degenerative diseases. We also included DEGs from other species such as mice, rats, and monkeys from the Aging Atlas that we converted to human genes by a form of homologous gene conversion. Users can enter a gene to quickly explore its changes in organ aging and various degenerative diseases. In addition, network diagrams and detailed DEG lists are used to help users quickly assess the relationship between genes in aging and degenerative diseases. This module will help scientists explore the potential molecular association between organ aging and diseases, and help develop early biomarkers and develop corresponding disease intervention strategies.

### DD gene enrichment analysis

The query strategies of most omics databases only support a single gene, which is not conducive to the joint query and analysis of multiple genes. Based on the collection of multiple degenerative disease signature genes constructed from single-cell transcriptomics and transcriptomics data, we developed an enrichment analysis method based on hypergeometric test (Huang et al., 2008). We then integrated this method into this module, which supports users to query multiple genes at the same time. Users can perform enrichment analysis to compare the input genes with all DEGs of degenerative diseases in the DD database. This capability is of great significance for helping users judge the association between gene sets of interest in various degenerative diseases and will help improve our understanding of organ degeneration.

### DD gene frequency analysis

Most degenerative diseases are accompanied by aging, which suggests that there are common molecular changes and mechanisms among degenerative diseases. Therefore, we developed a frequency analysis module to mine common molecular changes among multiple degenerative diseases. This module includes the most frequent DEGs and identifies the top 30 genes across different degenerative diseases. For DEGs in the transcriptome and single-cell transcriptome modules, genes with higher frequencies indicated their changes in multiple degenerative diseases, suggesting their potential value as molecular markers of organ degeneration more generally and as therapeutic targets. For example, the high frequency of *SPP1* (osteopontin) in single-cell datasets of various human degenerative diseases indicates its potential value as a disease biomarker. Furthermore, *CD74* (MHC HLA-DR gamma chain) showed a tendency to increase at both the single-cell and transcriptome levels, suggesting a possible widespread immune activation in organ degenerative diseases. In addition, this module demonstrates the association between the same gene and various degenerative diseases, enabling users to quickly retrieve the changes of a certain gene in various diseases.

### DD gene homologous analysis

Due to technical and ethical limitations associated with obtaining clinical samples, many studies of degenerative diseases are carried out in model organisms such as mice. Therefore, in the DD database, we also include datasets with mouse disease models, which increases the difficulty of gene conversion between species when counting high-frequency genes associated with diseases. Therefore, to facilitate the statistical analysis of high-frequency genes in the process of organ degeneration and degenerative diseases, we developed a separate homology analysis module. Users can assess disease high-frequency genes after realizing the homologous conversion of human and mouse genes. For example, the user selects human parameters, and the module converts all mouse genes to human genes based on homology. Users can browse key genes that function broadly across multiple datasets, providing biologists with a valuable resource for further experimental validation.

## Methods of database

### Omics data collection and processing

The transcriptome data, including gene expression count matrix and sample metadata, were downloaded from the public databases. The differential expression analysis was performed by DESeq2 1.32.0 with threshold *P*.adjust ≤ 0.05. The functional enrichment analysis of DEGs was performed by clusterProfiler 4.0.5.

The single-cell transcriptome data, including gene expression count matrix and cell type metadata, were downloaded from public databases, including GEO, ENA, and single cell portal. The differential expression analysis was performed by Seurat 4.0.5 with threshold *P*.adjust ≤ 0.05, |log_2_(foldchange)| ≥ 0.1 and min.pct ≥ 0.01 (Hao et al., 2021; Sun et al., 2022). The transcription factor analysis was predicted by SCENIC 1.2.4.

The spatial transcriptome data, including gene expression count matrix, spots metadata, and images, were downloaded from public databases. Low-quality spots with total count numbers less than 500 and genes less than 200 were filtered. The expression level of each gene was normalized by counts per million followed by log transformation.

The microbiome data include shot-gun metagenomics sequencing and amplicon sequencing data. Raw sequencing reads were downloaded from public databases. The metagenomics sequencing reads were quality-controlled by kneaddata v0.10.0 to filter reads aligned to the human reference genome hg19. The microbiome abundance table was quantified by the MetaPhlAn 3.0 with standard workflow. The amplicon sequencing data were performed with QIIME 2. Raw sequence data were quality filtered followed by denoising with DADA2. Taxonomy was assigned to amplicon sequence variants using the taxonomy classifier against the Greengenes (2022.10). The differential abundance analysis of both metagenomics and amplicon sequencing was performed by edgeR 3.34.0.

The epigenome data, including bigwig files, were downloaded from public databases. The proteome data were downloaded from public databases and literature.

### Web portal

The DD used SpringBoot web framework v2.5.9, and the front end of the server was developed with Vue 2.6.11 and Element UI 2.15.6. All data were stored in the MongoDB v4.2.0 database. The interactive visualization diagrams were implemented with the Echarts 5.0.2, igv.2.12.6, D3 7.4.4 and plotly 2.12.0.

## Concluding remarks

The aging process is accompanied by degeneration across multiple organs and is the major risk for degenerative diseases. A comprehensive omics database of degenerative disease stands to extend our understanding of molecular mechanisms of organ degeneration and inform the development of potential therapeutic or intervention targets for degenerative diseases. Hence, we constructed the DD, a multi-omics data resource platform for multiple degenerative diseases, to include the following salient features. (i) The DD database is currently the most comprehensive omics database for degenerative diseases, including 6 omics, 57 diseases, 165 datasets, and 3,519 samples. The DD database has interactive user-friendly data query and visualization tools, and all data can be downloaded with one click. (ii) The DD database provides a platform to conduct comparative analysis between different degenerative diseases and between different omics of the same disease, which promotes exploring the mechanisms of degenerative disease occurrence or progression and developing effective clinical treatments or intervention strategies for degenerative diseases. (iii) The DD database integrates aging-related omics data resources and indicates potential molecular relationships that may exist between aging and degenerative diseases. The DD database provides data resources for identifying novel aging or disease biomarkers, which promotes the realization of healthy aging. In the future, we are committed to advancing the DD database by updating omics sequencing data related to degenerative diseases, establishing a one-to-one correspondence between H&E staining images and spot expression levels for spatial gene expression data, refining data visualization techniques, and proactively incorporating user feedback through the DD portal.

## Data Availability

All data in DD is available at the website of Degeneration Directory to researchers. Users can directly download data without registration or login. The DD is available at http://data.iscr.ac.cn/degeneration/home.

## Abbreviations

AD: Alzheimer’s disease
ChIP-seq: chromatin immunoprecipitation sequencing
CSF: cerebro-spinal fluid
DD: degeneration directory
DEGs: differentially expressed genes
DNA: deoxyribonucleic acid
ENA: European nucleotide archive
GEO: gene expression omnibus
PD: Parkinson’s disease
rDNA: ribosomal deoxyribonucleic acid

## Code availability

All the custom codes are available from the corresponding authors upon reasonable request.
